# Prevalence of familial autoimmune diseases in juvenile idiopathic arthritis: results from the international Pharmachild registry

**DOI:** 10.1186/s12969-022-00762-y

**Published:** 2022-11-18

**Authors:** Joeri W. van Straalen, Sytze de Roock, Gabriella Giancane, Ekaterina Alexeeva, Elena Koskova, Pablo Mesa-del-Castillo Bermejo, Francesco Zulian, Adele Civino, Davide Montin, Nico M. Wulffraat, Nicolino Ruperto, Joost F. Swart, for the Paediatric Rheumatology International Trials Organisation (PRINTO)

**Affiliations:** 1 Department of Pediatric Immunology and Rheumatology, Wilhelmina Children’s Hospital, University Medical Center Utrecht, P.O. box 85090, 3508 AB Utrecht, the Netherlands; 2 Faculty of Medicine, Utrecht University, Utrecht, the Netherlands; 3 IRCCS Istituto Giannina Gaslini, Clinica Pediatrica e Reumatologia, Genoa, Italy; 4 Dipartimento di Neuroscienze, Riabilitazione, Oftalmologia, Genetica e Scienze Materno-Infantili (DiNOGMI), Università degli Studi di Genova, Genoa, Italy; 5 Federal State Autonomous Institution “National Medical Research Center of Children’s Health” of the Ministry of Health of the Russian Federation, Moscow, Russian Federation; 6 Federal State Autonomous Educational Institution of Higher Education, I.M. Sechenov First Moscow State Medical University of the Ministry of Health of the Russian Federation, Moscow, Russian Federation; 7 Department of Pediatric Rheumatology, National Institute of Rheumatic Diseases, Piestany, Slovakia; 8 Rheumatology, Pediatrics, Hospital Clínico Universitario Virgen de la Arrixaca, Murcia, Spain; 9 Department of Woman and Child Health, University of Padua, Padua, Italy; 10 UO Pediatria - Sez. Reumatologia e Immunologia pediatrica, P.O. “Vito Fazzi”, Lecce, Italy; 11 Immunology and Rheumatology Unit, Regina Margherita Children Hospital, Turin, Italy; 12 UOSID Centro trial, IRCCS Istituto Giannina Gaslini, Genoa, Italy

**Keywords:** Juvenile idiopathic arthritis, Familial autoimmune diseases, Pediatric rheumatology, Registry, Epidemiology

## Abstract

**Background:**

Little is known about the disposition to autoimmune diseases (ADs) among children diagnosed with JIA. In this study, we provide a comprehensive overview of the prevalence of and factors associated with ADs in parents of children with juvenile idiopathic arthritis (JIA).

**Methods:**

Prevalence rates of ADs and 95% Poisson confidence intervals were calculated for parents of JIA patients from the international Pharmachild registry and compared with general population prevalence rates as reported in the literature. Demographic, clinical and laboratory features were compared between JIA patients with and without a family history of AD using χ^2^ and Mann-Whitney *U* tests.

**Results:**

Eight thousand six hundred seventy three patients were included and the most common familial ADs were psoriasis, autoimmune thyroid disease, rheumatoid arthritis and ankylosing spondylitis. The prevalence of several ADs was higher in parents of the included JIA patients than in the general population. Clinical Juvenile Arthritis Disease Activity Scores at study entry and last follow-up were not significantly different between patients with (*n* = 1231) and without a family history of AD (*n* = 7442). Factors associated with familial AD were older age at JIA onset (*P* < 0.01), Scandinavian residence (*P* < 0.01), enthesitis-related arthritis, psoriatic arthritis and undifferentiated arthritis (*P* < 0.01), ANA positivity (*P* = 0.03) and HLA-B27 positivity (*P* < 0.01).

**Conclusions:**

Familial AD proves to be a risk factor for JIA development and certain diseases should therefore not be overlooked during family health history at the diagnosis stage. A family history of AD is associated with the JIA category but does not influence the severity or disease course.

**Supplementary Information:**

The online version contains supplementary material available at 10.1186/s12969-022-00762-y.

## Background

Juvenile idiopathic arthritis (JIA) is an umbrella term that comprises seven subtypes of arthritis of unknown cause that begin before the age of 16 years and last for more than 6 weeks [1]. Six out of seven subtypes are considered an autoimmune disease (AD), except for systemic JIA, which resembles more an autoinflammatory disease [1]. ADs are known to cluster within families and share common pathogenic mechanisms and genetic factors [2, 3]. However, little is known about the relationship between JIA and familial ADs. A previous study reported that 32% of 4677 JIA patients had at least one first-degree relative with an AD [4]. Furthermore, JIA patients with a family history of AD were reported to have higher disease activity and more often enthesitis-related arthritis (ERA) and psoriatic arthritis than JIA patients without such family history [5, 6]. Frequently described ADs in relatives of JIA patients are insulin-dependent diabetes mellitus (IDDM), JIA, rheumatoid arthritis (RA), autoimmune thyroid disease (AITD), spondyloarthropathy and psoriasis [4–11]. Nonetheless, the few studies about familial autoimmunity in JIA are either based on a limited number of patients, do not report prevalence rates within families or only report pre-selected ADs.

The objective of this study is to provide a comprehensive overview of the occurrence of and factors associated with ADs in parents of children with JIA from a large international registry [12] and to compare prevalence rates with those reported in the general population.

## Methods

### Patients

Patients were included from the international observational Pharmachild registry. Pharmachild started in 2011 with the objective of studying safety and effectiveness of drug therapies in JIA. Patients are included from Paediatric Rheumatology International Trials Organisation (PRINTO) centers from 31 countries worldwide. The registry includes patients with a diagnosis of JIA as per International League of Associations for Rheumatology (ILAR) criteria that are being treated with nonsteroidal anti-inflammatory drugs (NSAIDs), intraarticular steroids, systemic steroids, and/or conventional synthetic (cs-) or biological (b-) disease-modifying antirheumatic drugs (DMARD) as decided by the treating physician. Additional information about the Pharmachild registry is previously reported [12]. The extracted Pharmachild data were locked on 12 November, 2020. Patients without available information for family history of AD were excluded from the current study.

### Outcome and characteristics

Three researchers (JS, JvS and SdR) reviewed reported diseases in first degree relatives (i.e. mother and father) of the included JIA patients in order to ensure only definite diagnoses of ADs were included. Reported ADs were classified into the following categories: psoriasis, AITD, RA, ankylosing spondylitis, inflammatory bowel disease (IBD), JIA, asthma, IDDM, systemic lupus erythematosus, vitiligo, celiac disease, multiple sclerosis, uveitis, sarcoidosis, reactive arthritis, Sjögren’s syndrome, rheumatic fever, vasculitides, Still’s disease, familial Mediterranean fever, other autoimmune arthritis, other connective tissue disease and other AD. In addition, the following patient characteristics were collected: sex, geographic region, ethnicity, age at JIA onset, ILAR category of JIA, rheumatoid factor (RF) status, human leukocyte antigen (HLA) B27 status, antinuclear antibodies (ANA) status, number of active joints and clinical Juvenile Arthritis Disease Activity Score (cJADAS) at study entry and last visit and observation period (calculated from disease onset until last visit). Patients were grouped into the following geographic regions based on the country of the center in which they were treated: Western Europe, Central and Eastern Europe, Scandinavia, Northern Africa and the Middle East, Latin America and Southern Asia [13]. Ethnicity was reported at inclusion by the treating physician from a fixed set of categories. RF status was determined from two measurements at least 3 months apart according to ILAR criteria. Since not all patients had two available ANA tests, only the first test was used to determine ANA status. The cJADAS is a composite measure for disease activity that takes into account the number of active joints, physician global assessment of disease activity and parent/patient global assessment of well-being [14]. The latter two components of the cJADAS are measured on a 21-circle visual analogue scale ranging from 0 to 10 [15].

### Statistical analysis

Characteristics of patients with a family history of AD and those without were compared using the χ^2^ test for categorical variables and Mann-Whitney *U* test for numerical variables. Pairwise comparisons of categories of geographic region, ethnicity and ILAR subtypes were performed with Bonferroni correction. For each AD category, prevalence rates among parents and corresponding 95% Poisson confidence intervals were calculated. Prevalence rates of ADs in the general population were collected from the literature. For this, we included data from worldwide literature reviews or surveillance studies. ILAR categories of patients with different familial ADs were compared using the Fisher’s exact test. All comparative analyses were performed on complete cases and a *P*-value of < 0.05 was considered statistically significant. All analyses were performed with R version 4.0.0.

## Results

### Patient characteristics

At the cut-off date, a total of 9111 JIA patients were enrolled in Pharmachild, of which 438 (4.8%) had no available information about parental ADs and were excluded from further analyses. For the remaining cohort of 8673 JIA patients, the total observation period was 43,800 years with a median duration of 4.0 years (IQR: 1.8–7.3). The median duration from disease onset until study entry was 139 days (IQR: 55–458). The majority of patients were treated in European centers (*n* = 7590; 87.5%) (Table 1). An overview of the classification of treatment center countries into geographic regions is provided in an additional table (see Additional file 1). Of all included patients, 1231 (14.2, 95% CI: 13.5–14.9%) had a family history of AD. Out of these, 1107 (89.9%) had a family history of one AD, 116 (9.4%) had a family history of two ADs and 8 (0.6%) had a family history of three or more ADs. Patients with a family history of AD more often had ERA, psoriatic arthritis and undifferentiated arthritis than patients without a family history of AD (*P* < 0.01), were more often ANA (*P* = 0.03) and HLA-B27 positive (*P* < 0.01) and had an older age at JIA onset (*P* < 0.01). Patients without a family history of JIA more often had systemic arthritis. Furthermore, the proportion of patients from Scandinavia and Southern Europe was higher in patients with a family history of AD than in patients without such family history (*P* < 0.01). The same effect was observed for patients of European Caucasian and Northern African or Middle Eastern ethnicity (*P* < 0.01). No significant differences in sex, RF status and disease activity were observed.

**Table 1:** Characteristics of JIA patients with and without a family history of AD in parents (*n* = 8673)

Characteristic	No family history of AD (*n* = 7442)	Family history of AD (*n* = 1231)	*P*-value
Female, n (%)	5060 (68.0%)	847 (68.8%)	0.59
Age at JIA onset, median (IQR)	5.2 (2.4–9.8)	6.3 (2.5–10.8)	< 0.01*
Geographic region, n (%)			< 0.01*^a^
Central and Eastern Europe	2091 (28.1%)	210 (17.1%)	
Latin America	663 (8.9%)	50 (4.1%)	
Northern Africa and Middle East	157 (2.1%)	44 (3.6%)^b, c^	
Scandinavia	685 (9.2%)	177 (14.4%)^b, c^	
Southern Asia	153 (2.1%)	16 (1.3%)^d, e^	
Southern Europe	2384 (32.0%)	497 (40.4%)^b, c^	
Western Europe	1309 (17.6%)	237 (19.3%)^b, c, e^	
Ethnicity, n (%)			< 0.01*^a^
European Caucasian	5654 (85.6%)	1008 (89.1%)	
Hispanic	267 (4.0%)	21 (1.9%)^f^	
Indian	132 (2.0%)	12 (1.1%)	
Multiethnic	93 (1.4%)	14 (1.2%)	
Northern African or Middle Eastern	281 (4.3%)	60 (5.3%)^g^	
Southeast Asian	59 (0.9%)	6 (0.5%)	
Sub-Saharan African	75 (1.1%)	5 (0.4%)	
Other	47 (0.7%) *n* = 6608	5 (0.4%) *n* = 1131	
ILAR category, n (%)			< 0.01*^a^
ERA	777 (10.4%)	178 (14.5%)	
Oligoarthritis	2934 (39.4%)	319 (25.9%)^h^	
Polyarthritis RF-	2045 (27.5%)	228 (18.5%)^h^	
Polyarthritis RF+	335 (4.5%)	38 (3.1%)^h^	
Psoriatic arthritis	144 (1.9%)	146 (11.9%)^h, i, j, k^	
Systemic arthritis	898 (12.1%)	51 (4.1%)^h, i, j, l^	
Undifferentiated arthritis	309 (4.2%)	271 (22.0%)^h, i, j, k, m^	
Laboratory characteristics, n (%)			
ANA positive	2853 (40.9%) *n* = 6970	514 (44.3%) *n* = 1160	0.03*
RF positive)	356 (5.4%) *n* = 6629	49 (4.5%) *n* = 1088	0.26
HLA-B27 positive	865 (19.4%) *n* = 4467	229 (28.6%) *n* = 800	< 0.01*
Disease activity, median (IQR)			
Active joints at study entry	0 (0–2) *n* = 3143	0 (0–2) *n* = 583	0.18
cJADAS at study entry	2.0 (0.0–7.0) *n* = 2713	2.0 (0.0–7.0) *n* = 519	0.61
Active joints at last visit	0 (0–1) *n* = 3143	0 (0–1) *n* = 583	0.74
cJADAS at last visit	1.0 (0.0–5.0) *n* = 2713	1.0 (0.0–5.0) *n* = 519	0.82
Observation period in years, median (IQR)	4.0 (1.8–7.3)	3.9 (1.8–7.0)	0.24

*AD* Autoimmune disease, *ANA* Antinuclear antibodies, *cJADAS* Clinical JADAS, *ERA* Enthesitis-related arthritis, *HLA* Human leukocyte antigen, *IQR* Interquartile range, *ILAR* International League of Associations for Rheumatology, *JIA* Juvenile idiopathic arthritis, *RF* Rheumatoid factor

*P < 0.05, ^a^*P*-value indicates overall difference between categories, ^b^significantly different from Central and Eastern Europe, ^c^significantly different from Latin America, ^d^significantly different from Northern Africa and Middle East, ^e^significantly different from Scandinavia, ^f^significantly different from European Caucasian, ^g^significantly different from Hispanic, ^h^significantly different from ERA, ^i^significantly different from oligoarthritis, ^j^significantly different from polyarthritis RF-, ^k^significantly different from polyarthritis RF+, ^l^significantly different from psoriatic arthritis, ^m^significantly different from systemic arthritis

### Prevalence rates of familial ADs

**Fig. 1:**
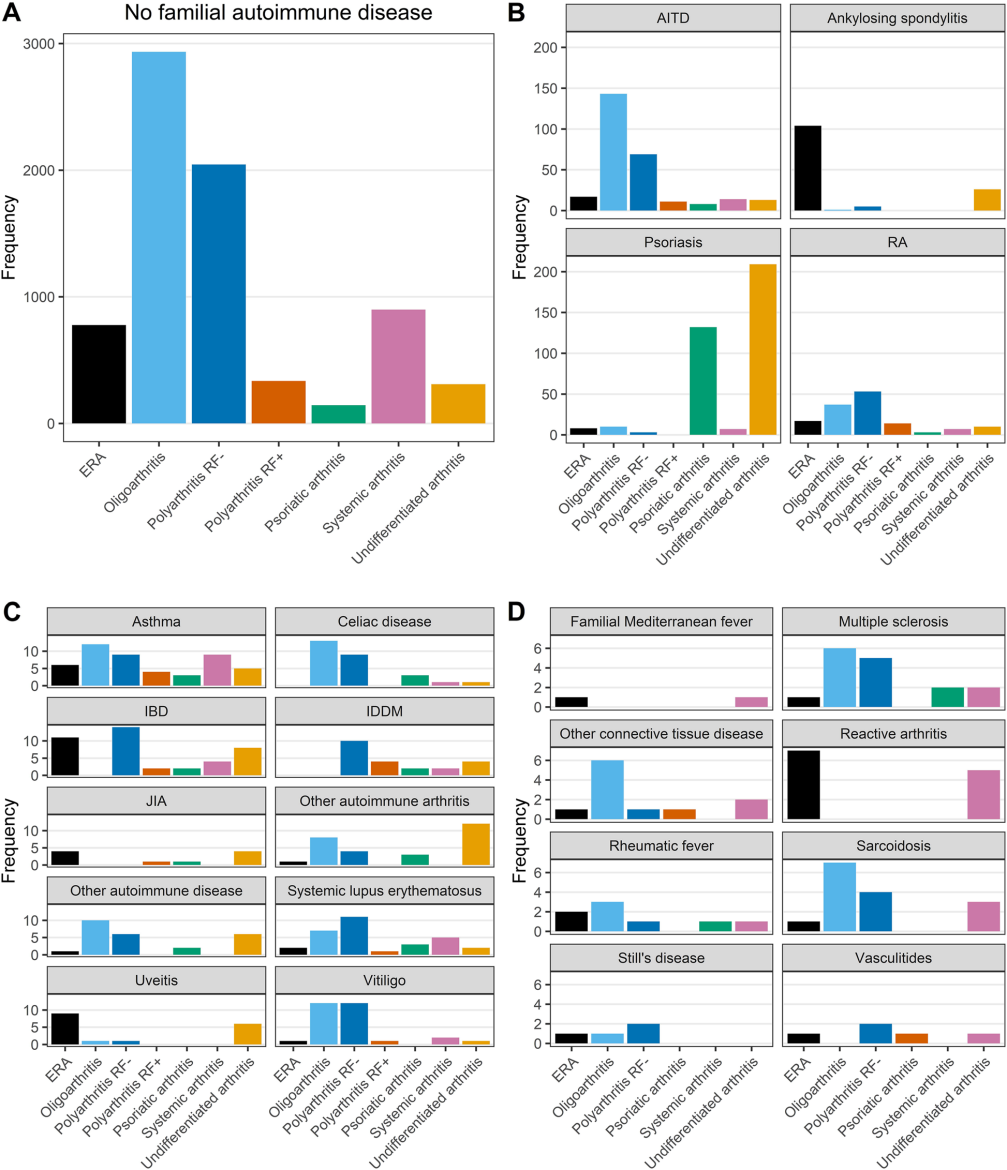
JIA categories of patients with and without familial autoimmune diseases. **A**: absolute frequencies of JIA categories for patients without familial autoimmune diseases. **B**-**D**: absolute frequencies of JIA categories for patients with different familial autoimmune diseases, each panel is displayed on a different scale. AITD: autoimmune thyroid disease, ERA: enthesitis-related arthritis, IBD: inflammatory bowel disease, IDDM: insulin-dependent diabetes mellitus, JIA: juvenile idiopathic arthritis, RA: rheumatoid arthritis, RF: rheumatoid factor

A total of 1366 ADs were reported in the parents (*n* = 17,346) of the included JIA patients. An overview of the classification of reported ADs is provided in an additional table (see Additional file 2). The most common diseases were psoriasis (*n* = 369; 2.1%), AITD (*n* = 275; 1.6%), RA (*n* = 141; 0.8%) and ankylosing spondylitis (*n* = 136; 0.8%). Prevalence rates of several ADs in parents of the included JIA patients were raised compared to reported prevalence rates in the general population, most notably ankylosing spondylitis, JIA and IDDM (Table 2). The observed prevalence of asthma and celiac disease was notably lower than the prevalence in the general population. The prevalence rates of separate diseases in the “other autoimmune disease” group are listed in an additional table (see Additional file 3). The distribution of ILAR categories amongst included JIA patients was significantly different for several familial ADs (Table 3). Of clinical relevance were the observations that patients with a family history of ankylosing spondylitis oftentimes had ERA or undifferentiated arthritis (*P* < 0.01), while patients with a family history of psoriasis oftentimes had psoriatic or undifferentiated arthritis (*P* < 0.01). For patients with different familial ADs, the absolute frequencies of ILAR categories are visualized in Fig. 1.

**Table 2:** Prevalence rates of ADs in parents of included JIA patients (n = 17,346)

Disease	Frequency	Prevalence per 100,000 (95% Poisson CI)	Global prevalence per 100,000
Psoriasis	369	2127 (1916 – 2356)	140–1990 [16]
Autoimmune thyroid disease	275	1585 (1404 – 1784)	GD:0–2000 [17] HT: 0–7000 [17]
Rheumatoid arthritis	141	813 (684–959)	300–700 [18]
Ankylosing spondylitis	136	784 (658–927)	20–350 [19]
Inflammatory bowel disease	68	392(304–497)	UC: 2–505 [20] CD: 1–322 [20]
Juvenile idiopathic arthritis	51	294 (219–387)	21 [21]
Asthma	48	277 (204–367)	4300 [22]
Insulin-dependent diabetes mellitus	38	219 (155–301)	39–96 [23]
Systemic lupus erythematosus	31	179 (121–254)	0–241 [24]
Vitiligo	29	167 (112–240)	100–1200 [25]
Other autoimmune arthritis	28	161 (107–233)	-^a^
Celiac disease	27	156 (103–226)	400–800 [26]
Other autoimmune disease	25	144 (93–213)	-^a^
Uveitis	17	98 (57–157)	9–730 [27]
Multiple sclerosis	16	92 (53–150)	2–164.6 [28]
Sarcoidosis	15	87 (48–143)	2–160 [29]
Reactive arthritis	12	69 (36–121)	0–200 [19]
Other connective tissue disease	11	63 (32–114)	-^a^
Sjögren’s syndrome	10	58 (28–106)	61 [30]
Rheumatic fever	8	46 (20–91)	-^b^
Vasculitides	5	29 (9–68)	-^a^
Still’s disease	4	23 (6–59)	1–6.8 [31]
Familial Mediterranean fever	2	12 (1–42)	-^c^

*AD* Autoimmune disease, *CD* Crohn’s disease, *CI* Confidence interval, *GD* Graves’ disease, *HT* Hashimoto’s thyroiditis, *UC* Ulcerative colitis

^a^heterogeneous group of diseases, ^b^not a chronic disease, ^c^mainly affects ethnic groups from the eastern Mediterranean region

**Table 3:** JIA categories of patients with a family history of different ADs

Family history of AD, n (%)	ERA (*n* = 955)	oJIA (*n* = 3253)	pJIA RF- (*n* = 2273)	pJIA RF + (*n* = 373)	psJIA (*n* = 290)	sJIA (*n* = 949)	uJIA (*n* = 580)	*P*-value
Psoriasis	8 (0.8%)	10 (0.3%)	3 (0.1%)	0 (0.0%)	129 (44.5%)	7 (0.7%)	205 (35.3%)	< 0.01*
AITD	17 (1.8%)	139 (4.3%)	69 (3.0%)	11 (2.9%)	8 (2.8%)	14 (1.5%)	13 (2.2%)	< 0.01*
RA	17 (1.8%)	37 (1.1%)	53 (2.3%)	14 (3.8%)	3 (1.0%)	7 (0.7%)	10 (1.7%)	< 0.01*
Ankylosing spondylitis	103 (10.8%)	1 (0.0%)	5 (0.2%)	0 (0.0%)	0 (0.0%)	0 (0.0%)	26(4.5%)	< 0.01*
IBD	11 (1.2%)	27 (0.8%)	14 (0.6%)	2 (0.5%)	2 (0.7%)	4 (0.4%)	8 (1.4%)	0.31
JIA	4 (0.4%)	17 (0.5%)	23 (1.0%)	1 (0.3%)	1 (0.3%)	0 (0.0%)	4 (0.7%)	0.01*
Asthma	6 (0.6%)	12 (0.4%)	9 (0.4%)	4 (1.1%)	3 (1.0%)	8 (0.8%)	5 (0.9%)	0.10
IDDM	0 (0.0%)	16 (0.5%)	10 (0.4%)	4 (1.1%)	2 (0.7%)	2 (0.2%)	4 (0.7%)	0.05*
SLE	2 (0.2%)	7 (0.2%)	11 (0.5%)	1 (0.3%)	3 (1.0%)	5 (0.5%)	2 (0.3%)	0.18
Vitiligo	1 (0.1%)	12 (0.4%)	12 (0.5%)	1 (0.3%)	0 (0.0%)	2 (0.2%)	1 (0.2%)	0.58
Other autoimmune arthritis	1 (0.1%)	8 (0.2%)	4 (0.2%)	0 (0.0%)	3 (1.0%)	0 (0.0%)	12(2.1%)	< 0.01*
Celiac disease	0 (0.0%)	13 (0.4%)	9 (0.4%)	0 (0.0%)	3 (1.0%)	1 (0.1%)	1 (0.2%)	0.08
Other autoimmune disease	1 (0.1%)	10 (0.3%)	6 (0.3%)	0 (0.0%)	2 (0.7%)	0 (0.0%)	6 (1.0%)	0.02*
Uveitis	9 (0.9%)	1 (0.0%)	1 (0.0%)	0 (0.0%)	0 (0.0%)	0 (0.0%)	6 (1.0%)	< 0.01*
Multiple sclerosis	1 (0.1%)	6 (0.2%)	5 (0.2%)	0 (0.0%)	0 (0.0%)	2 (0.2%)	2 (0.3%)	0.93
Sarcoidosis	1 (0.1%)	7 (0.2%)	4 (0.2%)	0 (0.0%)	0 (0.0%)	0 (0.0%)	3 (0.5%)	0.43
Reactive arthritis	7 (0.7%)	0 (0.0%)	0 (0.0%)	0 (0.0%)	0 (0.0%)	0 (0.0%)	5 (0.9%)	< 0.01*
Other connective tissue disease	1 (0.1%)	6 (0.2%)	1 (0.0%)	0 (0.0%)	1 (0.3%)	0 (0.0%)	2 (0.3%)	0.23
Sjögren’s syndrome	1 (0.1%)	5 (0.2%)	0 (0.0%)	0 (0.0%)	2 (0.7%)	1 (0.1%)	1 (0.2%)	0.07
Rheumatic fever	2 (0.2%)	3 (0.1%)	1 (0.0%)	0 (0.0%)	0 (0.0%)	1 (0.1%)	1 (0.2%)	0.65
Vasculitides	1 (0.1%)	0 (0.0%)	2 (0.1%)	0 (0.0%)	1 (0.3%)	0 (0.0%)	1 (0.2%)	0.07
Still’s disease	1 (0.1%)	1 (0.0%)	2 (0.1%)	0 (0.0%)	0 (0.0%)	0 (0.0%)	0 (0.0%)	0.80
FMF	1 (0.1%)	0 (0.0%)	0 (0.0%)	0 (0.0%)	0 (0.0%)	0 (0.0%)	1 (0.2%)	0.11

In case both parents of one JIA patient have a history of the same AD, the total number of AD cases per row does not add up to the numbers listed in Table 2

*AD* Autoimmune disease, *AITD* Autoimmune thyroid disease, *ERA* Enthesitis-related arthritis, *FMF* Familial Mediterranean fever, *IBD* Inflammatory bowel disease, *IDDM* Insulin-dependent diabetes mellitus, *JIA* Juvenile idiopathic arthritis, *oJIA* Oligoarthritis, *pJIA* Polyarthritis, *RA* Rheumatoid arthritis, *RF* Rheumatoid factor, *psJIA* Psoriatic arthritis, *sJIA* Systemic arthritis, *SLE* Systemic lupus erythematosus, *uJIA* Undifferentiated arthritis

*P < 0.05

## Discussion

The objective of this study was to present prevalence rates of ADs in parents of JIA patients and identify factors associated with such family history. According to our study, the prevalence rates of several ADs in parents of JIA patients are higher than those in the general population, with the most frequent familial ADs in JIA being psoriasis, AITD, RA and ankylosing spondylitis. Factors associated with a family history of AD in JIA are the geographic region, ethnicity, age at JIA onset, ILAR category, ANA status and HLA-B27 status.

In this study, the observed proportion of JIA patients with a family history of AD in first-degree relatives (14.2, 95% CI: 13.5–14.9%) was lower than in previous studies (21.4–31.8%) [4, 11]. This is possibly explained by the method of reporting familial autoimmunity in Pharmachild, differences in the target population and/or the definition of first-degree relatives. In Pharmachild, familial ADs are registered using self-reporting by the patient or parent, which might have led to an underestimation of the absolute prevalence of familial AD in JIA. Pharmachild furthermore defines a first-degree relative as the mother or father of a patient, whereas other definitions also include siblings. In addition, Pharmachild captures whether or not first degree relatives of JIA patients have a history of AD but does not distinguish between mother and father. Because of this, we could not report an overall prevalence of AD in parents since one parent can have a history of multiple ADs. Based on the number of JIA patients with a family history of AD (*n* = 1231) in parents (*n* = 17,346) and the total number of reported ADs (*n* = 1366), the overall prevalence of AD in parents would have to be little over 7.1% for the current study. This number is still higher than the reported prevalence of AD in the general population of nearly 5% [2, 3, 32], which is in accordance with previous studies [7, 11, 33, 34]. To our knowledge, no overall prevalence rate for AD has yet been reported for parents of JIA patients.

We observed several differences in characteristics of JIA patients with and without a family history of AD. A small study by Tronconi et al [8] did not find an association between a family history of AD and the subtype and age at onset of JIA. On the contrary, the current study observed that patients with psoriatic arthritis, undifferentiated arthritis and ERA reported relatively often a family history of AD as opposed to patients with systemic arthritis and oligoarthritis, which is also in line with two previous studies [4, 6]. The association between HLA-B27 and a family history of AD corresponds with the observed effect for JIA category, given that, 73.7% of ERA patients and 38.3% of undifferentiated arthritis patients in our study were HLA-B27 positive. The association of familial AD with psoriatic arthritis and ERA can be explained by the ILAR criteria of these categories, which includes a family history of psoriasis for psoriatic arthritis, and a family history of ankylosing spondylitis, ERA, sacroiliitis with IBD, Reiter’s syndrome or acute anterior uveitis for ERA [35]. Also, the high frequency of familial AD in the undifferentiated arthritis group is likely due to the fact that many JIA patients are assigned to this group because of a family history of psoriasis, which serves as an exclusion criterion for all other JIA categories except psoriatic arthritis. It has previously been discussed whether or not this exclusion criterion should be revised [36]. Indeed, we observed that a family history of psoriasis was relatively common for patients with psoriatic and undifferentiated JIA and a family history of ankylosing spondylitis and uveitis for ERA. It was furthermore interesting to see that a family history of AITD was relatively common in JIA patients with oligoarthritis, given that several studies have reported a link between oligoarthritis and AITD in JIA [33, 34, 37, 38]. On the other hand, a family history of AD was negatively associated with systemic arthritis in the current study. This can be explained by the autoinflammatory instead of autoimmune nature of this JIA category [1]. Previous studies have reported contradictory relationships between the age at JIA onset and a family history of AD [5, 6, 8, 39–41]. In this study, familial AD was associated with older age at JIA onset. It is unclear what causes this effect, but it might be confounded by the category of JIA since oligoarthritis commonly presents at a young age and ERA during late childhood [42]. We furthermore found that familial AD was associated with ANA positivity in the included JIA patients while previous studies report opposing results [5, 39, 40]. These differences might be due to the number and type of familial ADs investigated in each study. ANA are a marker of several ADs including AITD [43], which was a frequently reported AD in the parents of the included JIA patients. We also observed a statistically significant difference in the distribution of geographic regions. Patients from Scandinavia, Southern Europe, Western Europe, Northern Africa and the Middle East had relatively more often a family history of AD compared to patients from Central and Eastern Europe, Latin America and Southern Asia. The same effect was observed for patients of European Caucasian and Northern African or Middle Eastern ethnicity compared to patients of other ethnicities. These findings largely support existing epidemiological data on the worldwide distribution of AD, with higher relative frequencies in industrialized countries compared to developing countries [44]. Therefore, at the diagnosis stage of possible JIA, physicians might want to ask about a family history of AD especially in children of the before mentioned ethnicities with relatively increased prevalence. In the current study, we found no effect of familial autoimmunity on (the course of) disease activity in the included JIA patients. Previously, two studies reported that JIA patients with a family history of AD had higher disease activity and longer active disease duration than JIA patients without such family history [5, 6]. This contradiction might be a result of differences in the target population and study design, given that one of the mentioned studies included a highly consanguineous JIA population from Saudi Arabia and the other study only included JIA patients from Iran in a case-control design. Nevertheless, other studies indicate a more severe disease course and unfavorable outcome for psoriatic arthritis patients compared to other JIA categories [45, 46]. Also, it has been reported that a family history of AD is associated with the development of comorbidities in JIA [47, 48], which was beyond the scope of the current study.

Amongst others, psoriasis, RA, ankylosing spondylitis, JIA, IDDM and Still’s disease were more prevalent in parents of the included JIA patients than in the general population based on the available literature. Familial AD therefore proves to be a risk factor for JIA development. In a study of Finnish JIA patients, Pohjankoski et al. also observed higher prevalence rates of RA, spondyloarthropathy, psoriatic arthritis, JIA and IDDM in parents and full siblings of JIA patients compared to the general population [11]. The population prevalence rates for AITD reported in the literature varied to a large extent, most definitely due to differences in diagnostic criteria. Therefore, in the present study we could not conclude with certainty if AITD is more common in parents of JIA patients than in the general population. Nevertheless, a previous study reported that the prevalence of Hashimoto’s thyroiditis in first and second-degree relatives was significantly higher for JIA patients compared to age-matched healthy controls [7]. All these findings are consistent with the hypothesis that clinically distinct ADs share common genetic susceptibility factors [4]. On the other hand, the prevalence of asthma and celiac disease in our data was decreased compared to the reported general population prevalence rates, perhaps due to the self-reporting mechanism of capturing familial autoimmunity data in Pharmachild. As an example, many children outgrow asthma [49] and might therefore not report this disease in adulthood. Pohjankoski et al. also reported no increased frequency of celiac disease in families of JIA patients compared to the general population [11].

Our study has a few limitations. First, it is likely that the absolute prevalence of familial autoimmunity in JIA is underestimated by our data since these were gathered using self-reporting by the patients and their parents, as described previously. In order to minimize the probability of recall bias, we focused only on parents and did not include ADs in second and third-degree relatives. Secondly, the majority of included patients were treated in European centers, which might also have influenced prevalence rates of familial ADs. Lastly, since it was not possible to distinguish between male or female parents in our data, we could not study a possible parent-of-origin effect. A previous study has reported that the prevalence of ADs among mothers of JIA patients was significantly higher than that of fathers, suggesting a maternal parent-of-origin effect wherein the sex of the parent with an AD influences the expression of JIA in offspring [50].

Nevertheless, we present the largest study on familial AD in JIA so far. We included patients from multiple countries around the world, making it possible to study geographical differences in prevalence rates. We furthermore present information for all reported familial ADs in parents of JIA patients from the Pharmachild registry, and did not focus on a pre-selected set of diseases. This study confirms previously reported associations with familial AD in JIA and demonstrates that a family history of AD is not related to the disease course. These study results might provide useful information for pediatric rheumatologists at the diagnosis stage of a child with (possible) JIA.

## Conclusions

In conclusion, we provide for the first time a comprehensive overview of the frequency of different ADs in parents of JIA patients. Several of these diseases have an increased prevalence compared to the general population. Psoriasis, AITD, RA and ankylosing spondylitis were most often reported and should therefore not be overlooked during family health history at the diagnosis stage of a child with possible JIA. A family history of AD is particularly associated with psoriatic arthritis, undifferentiated arthritis and ERA but does not influence the severity or course of JIA.

## Supplementary Information

**Additional file 1.** Classification of treatment center countries of JIA patients into geographic regions.

**Additional file 2.** Classification of reported autoimmune diseases in parents of included JIA patients.

**Additional file 3. **Prevalence rates of diseases from the “other autoimmune disease” category in parents of included JIA patients (*n* = 17,346).

## Data Availability

All relevant data are reported in the article. Additional details can be provided by the corresponding author upon reasonable request. The Pharmachild registry is registered at Clinicaltrials.gov (NCT01399281) and at the European Network of Centres for Pharmacoepidemiology and Pharmacovigilance (ENCePP; http://www.encepp.eu/encepp/viewResource.htm?id=19362).

## Abbreviations

AD: Autoimmune disease
ANA: Antinuclear antibodies
CD: Crohn’s disease
CI: Confidence interval
cJADAS: Clinical JADAS
ERA: Enthesitis-related arthritis
GD: Graves’ disease
HLA: Human leukocyte antigen
HT: Hashimoto’s thyroiditis
IQR: Interquartile range
IBD: Inflammatory bowel disease
IDDM: Insulin-dependent diabetes mellitus
ILAR: International League of Associations for Rheumatology
JIA: Juvenile idiopathic arthritis
oJIA: Oligoarthritis
pJIA: Polyarthritis
psJIA: Psoriatic arthritis
RA: Rheumatoid arthritis
RF: Rheumatoid factor
sJIA: Systemic arthritis
SLE: Systemic lupus erythematosus
UC: Ulcerative colitis
uJIA: Undifferentiated arthritis
