# Supplementary material for: Prevalence of familial autoimmune diseases in juvenile idiopathic arthritis: results from the international Pharmachild registry
Source: Pediatr Rheumatol Online J. 2022 Nov 18;20:103. doi: 10.1186/s12969-022-00762-y (PMC9673358; doi:10.1186/s12969-022-00762-y)
Supplement: Supplementary file 1 — Additional file 1. Classification of treatment center countries of JIA patients into geographic regions. [file 12969_2022_762_MOESM1_ESM.docx]

**Additional file 1. Classification of treatment centre countries of JIA patients into geographic regions.**

| Geographic region | Country |
| --- | --- |
| Western Europe | Austria, France, the Netherlands and Switzerland |
| Southern Europe | Greece, Italy and Spain |
| Central and Eastern Europe | Bulgaria, Croatia, Czech Republic, Hungary, Latvia, Lithuania, Poland, Romania, Serbia, Slovakia and Russia |
| Scandinavia | Denmark and Norway |
| Northern Africa and the Middle East | Israel, Libya, Oman, Saudi Arabia and Turkey |
| Latin America | Argentina, Brazil, Ecuador and Mexico |
| Southern Asia | India and Singapore |
