# Supplementary material for: Prevalence of familial autoimmune diseases in juvenile idiopathic arthritis: results from the international Pharmachild registry
Source: Pediatr Rheumatol Online J. 2022 Nov 18;20:103. doi: 10.1186/s12969-022-00762-y (PMC9673358; doi:10.1186/s12969-022-00762-y)
Supplement: Supplementary file 2 — Additional file 2. Classification of reported autoimmune diseases in parents of included JIA patients. [file 12969_2022_762_MOESM2_ESM.docx]

**Additional file 2. Classification of reported autoimmune diseases in parents of included JIA patients.**

| Autoimmune disease category | Reported autoimmune diseases included |
| --- | --- |
| Psoriasis | Psoriasis |
| Autoimmune thyroid disease | Hashimoto’s thyroiditis, Graves’ disease |
| Rheumatoid arthritis | Rheumatoid arthritis |
| Ankylosing spondylitis | Ankylosing spondylitis |
| Inflammatory bowel disease | Crohn’s disease, ulcerative colitis |
| Juvenile idiopathic arthritis | Juvenile idiopathic arthritis |
| Asthma | Asthma |
| Insulin-dependent diabetes mellitus | Insulin-dependent diabetes mellitus |
| Systemic lupus erythematosus | Systemic lupus erythematosus |
| Vitiligo | Vitiligo |
| Celiac disease | Celiac disease |
| Multiple sclerosis | Multiple sclerosis |
| Uveitis | Uveitis |
| Sarcoidosis | Sarcoidosis |
| Reactive arthritis | Reactive arthritis |
| Sjögren’s syndrome | Sjögren’s syndrome |
| Rheumatic fever | Rheumatic fever |
| Vasculitides | ANCA-associated vasculitis, Churg-Strauss syndrome, giant cell arteritis, Henoch-Schönlein purpura, leukocytoclastic vasculitis, microscopic polyangiitis, nodular vasculitis, polyarteritis nodosa, Takayasu’s arteritis, Wegener’s granulomatosis |
| Still’s disease | Still’s disease |
| Familial Mediterranean fever | Familial Mediterranean fever |
| Other autoimmune arthritis | Psoriatic arthritis, undifferentiated arthritis, unspecified arthritis |
| Other connective tissue disease | Scleroderma, mixed connective tissue disease |
| Other autoimmune disease | Eczema, alopecia areata, cutaneous lupus, immune thrombocytopenia, pemphigus, antiphospholipid antibody syndrome, autoimmune nephritis, autoimmune atrophic gastritis, autoimmune hemolytic anemia, autoimmune hepatitis, Evans syndrome, miastenia, dermatomyositis |
