# Supplementary material for: Prevalence of familial autoimmune diseases in juvenile idiopathic arthritis: results from the international Pharmachild registry
Source: Pediatr Rheumatol Online J. 2022 Nov 18;20:103. doi: 10.1186/s12969-022-00762-y (PMC9673358; doi:10.1186/s12969-022-00762-y)
Supplement: Supplementary file 3 — Additional file 3. Prevalence rates of diseases from the “other autoimmune disease” category in parents of included JIA patients (n = 17,346). [file 12969_2022_762_MOESM3_ESM.docx]

**Additional file 3. Prevalence rates of diseases from the “other autoimmune disease” category in parents of included JIA patients (n = 17,346).**

| Disease | Frequency | Prevalence per 100,000 (95% Poisson CI) |
| --- | --- | --- |
| Eczema | 6 | 34.6 (12.5 – 75.5) |
| Alopecia areata | 3 | 17.3 (3.3 – 50.9) |
| Cutaneous lupus | 3 | 17.3 (3.3 – 50.9) |
| Immune thrombocytopenia | 2 | 11.5 (1.1 – 42.0) |
| Pemphigus | 2 | 11.5 (1.1 – 42.0) |
| Antiphospholipid antibody syndrome | 2 | 11.5 (1.1 – 42.0) |
| Autoimmune nephritis | 1 | 5.8 (0.0 – 32.7) |
| Autoimmune atrophic gastritis | 1 | 5.8 (0.0 – 32.7) |
| Autoimmune hemolyticanemia | 1 | 5.8 (0.0 – 32.7) |
| Autoimmune hepatitis | 1 | 5.8 (0.0 – 32.7) |
| Evans syndrome | 1 | 5.8 (0.0 – 32.7) |
| Miastenia | 1 | 5.8 (0.0 – 32.7) |
| Dermatomyositis | 1 | 5.8 (0.0 – 32.7) |
